# Alcohol and Relatively Pure Cannabis Use, but Not Schizotypy, are Associated with Cognitive Attenuations

**DOI:** 10.3389/fpsyt.2014.00133

**Published:** 2014-09-29

**Authors:** Daniela A. Herzig, David J. Nutt, Christine Mohr

**Affiliations:** ^1^Department of Experimental Psychology, University of Bristol, Bristol, UK; ^2^Institute for Response-Genetics, University of Zurich, Kilchberg, Switzerland; ^3^Clienia AG Littenheid, Littenheid, Switzerland; ^4^Neuropsychopharmacology Unit, Imperial College London, London, UK; ^5^Faculté des Sciences Sociales et Politiques, Institut de Psychologie, Université de Lausanne, Lausanne, Switzerland

**Keywords:** polydrug use, licit drug use, cognition, schizotypy, psychosis-proneness

## Abstract

Elevated schizotypy relates to similar cognitive attenuations as seen in psychosis and cannabis/polydrug use. Also, in schizotypal populations cannabis and polydrug (including licit drug) use are enhanced. These cognitive attenuations may therefore either be a behavioral marker of psychotic (-like) symptoms or the consequence of enhanced drug use in schizotypal populations. To elucidate this, we investigated the link between cognitive attenuation and cannabis use in largely pure cannabis users (35) and non-using controls (48), accounting for the potential additional influence of both schizotypy and licit drug use (alcohol, nicotine). Cognitive attenuations commonly seen in psychosis were associated with cannabis and alcohol use, but not schizotypy. Future studies should therefore consider (i) non-excessive licit substance use (e.g., alcohol) in studies investigating the effect of cannabis use on cognition and (ii) both enhanced illicit and licit substance use in studies investigating cognition in schizotypal populations.

## Introduction

*Cannabis sativa* (marijuana) is currently the most widely used illegal substance in Europe (1). Past year cannabis use was reported by about 11.2% of all 15–34 year olds (1). This elevated prevalence rate (when compared to other illicit drug use) is concerning, because cannabis use might go along with both cognitive attenuation (2, 3) and mental health problems, in particular psychosis (4–9). Yet, only a minority of cannabis users (CU) will develop psychotic illnesses (5–7, 10). Therefore, other factors likely influence adverse consequences associated with cannabis use (9, 11).

Here, we focused on the supposedly negative implications of cannabis use on cognitive functioning while accounting for individuals’ schizotypy and associated licit drug use. We did so based on the following reasoning. On the one hand, relatively pure CU (i.e., no regular drug use other than marihuana, cigarettes, or alcohol) have attenuated cognitive functioning compared to non-users (3), e.g., in verbal working memory (12), verbal short-term memory (13), and mental flexibility (3, 14). On the other hand, as detailed below, schizotypal personality features are not only part of the psychosis dimension but also associate with cognitive attenuations, cannabis use, as well as licit drug use.

The schizotypy approach assumes that psychotic symptoms exist along a continuum, with severest symptoms occurring in schizophrenia and mild sub-clinical ones in schizotypal individuals from the general population (15). Schizotypy is commonly assessed using self-report questionnaires (16, 17). Scores on these questionnaires can be commonly divided into symptom dimensions known from patients, e.g., consisting of positive schizotypy (e.g., magical thinking, unusual experiences), negative schizotypy (e.g., anhedonia), and cognitive disorganization [e.g., odd speech and behavior; (16, 18)]. When it comes to laboratory measures, high as compared to low schizotypes yield relatively impaired cognitive performance, e.g., in working memory (19, 20), cognitive flexibility (21), and verbal short-term memory (22, 23). Most relevant to our reasoning, high schizotypy goes along with elevated substance use of, e.g., cannabis (24–28), nicotine, and alcohol (26, 29). Similarly, an elevated drug use has been reported in schizophrenia when compared to healthy controls (2, 6, 30–34).

Given the above described interrelationships, it is possible that the link between cannabis and cognition is influenced by individuals’ schizotypal features and/or additional licit drug use. The latter reasoning is particularly likely given that CU show higher consumption of nicotine and/or alcohol when compared to non-users (35, 36). Studies that assessed all three variables (cognition, cannabis use, and schizotypy) found that CU showed both worse cognitive performance and higher schizotypy scores (24, 37), and that only in CU schizotypal symptoms correlated with worse cognitive performance (37). When it comes to licit drug use, the available information is even scarcer, as these studies did not report on a potential effect of licit (nicotine, alcohol) drug use (24, 37). We therefore investigated the link between cognitive attenuation and cannabis use in largely pure CU and non-cannabis users (nCU), accounting for the potential additional influence of both schizotypy and licit drug use (alcohol, nicotine).

We expected that both illicit and licit drug use might be more important than schizotypy to explain variance in cognitive performance (38–40). If schizotypy would additionally or instead explain variance in cognitive performance, we would expect the cognitive disorganization dimension (25–27) and/or positive schizotypy dimension (24, 41, 42) to be more relevant than the negative schizotypy dimension (27, 37, 42–44) that frequently resulted in heterogeneous findings.

## Materials and Methods

### Participants

We recruited participants via advertisements looking for both pure CU (see also screening section) and non-nicotine consuming nCU. Advertisements were distributed at the local University and its vicinity on paper and electronically. We also used a local website (“Gumtree”). We recruited 83 healthy native English-speaking participants [35 CU (23 males) and 48 nCU (20 males)]. Participants either received monetary compensation for travel expenses or course credits. The University of Bristol ethics committee approved this study. All participants provided written informed consent prior participation.

### Screening

In both groups (CU, nCU), people were excluded if they reported excessive alcohol use (>50 units of alcohol/week for men, >35 units of alcohol/women), alcohol use within 12 h prior to testing, a neurological, psychological, or psychiatric history, or visual problems (including dyslexia). Prior to study inclusion, participants were alerted that we would ask for a urine sample for drug screening. We then asked about illegal substance use within the past 3 months. To encourage honest responding, volunteers were kept unaware of the drug spectrum assessed with the urine test (it detected cannabis metabolites until about 2 weeks after its consumption). To ensure recruitment of largely pure CU, participants were excluded if they indicated regular illicit drug use (apart from cannabis) in the past 3 months (more than twice) and/or use within 2 weeks prior testing. Participants were also asked about their cannabis and nicotine use habits (e.g., average amount of times cannabis used/cigarettes per week) in the past 30 days prior testing. Data with a negative drug test were not excluded if CU self-reported occasional use (on average 1–2 times/week within the past 30 days), and/or indicated regular or frequent use (on average >2 times/week in the past 30 days), but not within the past 2 weeks (45). If regular or frequent CU indicated use within the past 2 weeks, participants with a negative drug test were excluded from further analysis. The healthy nCU were excluded if they reported nicotine and cannabis use in the past 30 days, and if they showed a positive drug test.

### Procedure

Participants were firstly screened by means of the procedure outlined above. Subsequently, participants came to the local department for a 1 h testing session. The CU were asked to abstain prior testing from (i) cannabis use at least 2 h and (ii) nicotine use 30 min. We chose this abstinence period for cannabis, because general psychotropic effects seem to taper off 2–3 h post-consumption (46). In the case of nicotine, acute effects on cognition seem observable within 15–35 min of nicotine consumption [e.g., Ref. (47, 48)]. After having provided written informed consent, participants underwent the urine test before continuing to the testing session. Participants first completed the drug and schizotypy questionnaires, before performing the cognitive tasks. Participants performed the tasks outlined below as well as a handedness questionnaire and a lateralized lexical decision task. Results from the latter will be presented elsewhere. Task order was randomized between participants. Finally, participants were debriefed and reimbursed for their time.

### Questionnaires

#### Schizotypy

The Oxford-Liverpool Inventory of Feelings and Experiences (17, 49) is a 159-item self-report instrument consisting of the following sub-scales: positive schizotypy (unusual experiences = UnEx, 30 items), negative schizotypy (Introvertive Anhedonia = IntAn, 27 items), and cognitive disorganization (= CogDis, 24 items). Finally, 23 items assess impulsive non-conformity (ImpNC). Normative values can be found in Mason et al. (17, 49). We did not account for IntAn and ImpNC in this study, because of the heterogeneity in findings in the former case (see Introduction) and because ImpNC does not represent a schizotypy dimension (17).

#### Drug questions

Participants reported on their prior drug use (lifetime, past year, and past month drug use), e.g., their alcohol, cigarette, cannabis, cocaine, amphetamine, hallucinogen, opiate, and prescribed drug use. Items were taken from the national household survey on drug abuse (50). This questionnaire taps into seven DSM-IV criteria for drug dependence (past 12 months) by asking if people (1) spent a lot of time obtaining, using, or recovering from the drug, (2) experienced a marked increase in amount and frequency of drug use, (3) experienced a marked decrease in the drug effect, and (4) gave up or reduced important social, occupational, or recreational activities due to drug use. It also asks if people experienced (5) drug-induced psychological problems (such as depressive mood), (6) drug-induced physical problems, and (7) a persistent desire for the drug or unsuccessful attempts to stop drug use. For each positive answer, participants received 1 point (maximum score 7) with higher values indicating higher substance use severity. Participants also indicated the average amount of joints/cigarettes per week they used within the past 30 days. For this reason, it is possible that current non-smokers (no tobacco use within the past month) receive a nicotine severity score ≠ 0 if they have smoked within the past 12 months (this only concerned 2 of the 48 non-smokers).

### Behavioral measures: cognitive functioning

#### Trail making task

The trail making task (TMT) assessed executive functioning (51, 52). In the TMT A, participants connected numbered circles in chronological order (1–25) by drawing a line, as fast as possible. In the subsequent TMT B, participants saw circles containing numbers or letters. They drew a line as quickly as possible in chronological order switching between numbers and letters, i.e., from 1 to A, from A to 2, from 2 to B, etc. The RT for both versions was recorded. The TMT-index (RT version B minus version A) was used as an estimate of cognitive flexibility (53). Norm values are available from Tombaugh (54).

#### Verbal short-term memory (story-recall/logical memory)

We used a subtest of the revised version of the Wechsler adult intelligence scale (55). The experimenter read out a 60 words story. The participant was asked immediately afterwards to recall as many details as possible (maximum of 23 possible points). Normative data for young adults and university samples can be found in Bowden et al. (56) and Ivison (57), respectively.

#### Verbal working memory (two-back task)

Comparable to previous reports (58, 59), participants saw 64 sequentially presented digits (ranging from 1 to 9) in the middle of the computer screen (white on black background, font Arial, size 16). Participants had to press a given response key when the current digit (*n*) was identical to the digit *n*-2 (target trials). In all non-target trials, participants had to press another response key. Response key allocation was counterbalanced between participants. A third of the trials (*n* = 20) were target trials, and the remaining trials were non-target trials [*n* = 44; e.g., 59]. To increase task difficulty, we added intrusion trials. These were included to prevent restarting memorization after each successful target identification. Consequently, targets could occur twice in a row. Each stimulus appeared for 2000 ms, with an inter-stimulus interval of 500 ms (60) before the next digit appeared. Participants had to respond within 2500 ms, otherwise the trial was counted as an omission. All participants performed 16 practice trials. We measured the percentage of the correctly identified targets, as well as mean RTs for correct trials (59, 61).

As an additional note, we also measured a computerized Go NoGo task. Due to an overall ceiling performance, we omitted this task from further analysis.

### Data analysis

To determine if cannabis use affects cognitive functioning, we conducted separate univariate ANOVAs with group (CU, nCU) as between-subjects factor on the following measures: percentage of correct responses [(number of correctly identified target stimuli × 100)/total of targets] and RT in the two-back task, TMT-index, and the percentage of correctly identified units in the story-recall task [(number of correctly identified units × 100)/total of units)].

To determine effects of drug use and schizotypy on cognition, we firstly investigated the demographic characteristics of our population. We found sex differences between drug groups (see Results for details). We then correlated all variables with the outcome measures to preselect variables for the regression model (see Results for details). Neither age nor schizotypy significantly correlated with the cognitive measures. Due to the previous literature (see Introduction), we nevertheless kept UnEx scores and CogDis scores for the hierarchical regressions as follows: sex was entered in the first step, schizotypy (UnEx scores, CogDis scores) in the second step, and drug use severity (nicotine, alcohol, and cannabis) in the third step. Severity was preferred over frequency due to the former measure’s relevance to clinical addiction. Exploratory analysis confirmed that drug use severity was more important than drug use frequency in the current regression analyses. Thus, three blocks of predictors were entered in nested blocks, meaning that each subsequent block contained all prior predictors and the additional predictors from the current block. Presentation of results only includes the new predictors entered, for economy of presentation. All tolerance values were above 0.2 (62) and all independent variables were mean-centered. Thus, multi-collinearity between the independent variables was considered negligible. The dependent variables were (i) percentage correctly identified targets and mean RT for correctly identified targets and non-targets in the two-back task; (ii) TMT-index; and (iii) percentage of correctly recalled units in the story-recall task.

Kolmogorov–Smirnov tests for the groups separately revealed normal distribution for all behavioral measures. All *p*-values were two-tailed and the α-level was set at 0.05.

## Results

### Participants

We identified 35 CU (out of 83 healthy native English-speakers). On average (±SD), CU smoked 11.14 joints per week (±12.16), a frequency that can be classified as heavy use [>5 joints per week; (63)]. The last cannabis consumption was on average more than 24 h ago (114.37 ± 143.02 h) with 4 CU reporting cannabis use 2–6 h before testing. When only individuals are considered whose last cannabis consumption was more than 6 h ago, the results stayed largely the same. Age of cannabis use onset was at 15.46 years of age (±1.87 years). Within the CU group, 13 individuals were educated to college level (37%), 1 to secondary school (3%), and 21 to university degrees (60%). Of the 48 nCU, 12 individuals had college degrees (25%) and 36 had university degrees (75%). A chi-square test indicated that the two groups did not differ from each other in terms of highest finished education level [χ^2^(df = 2) = 3.03, *p* = 0.22]. A chi-square test on sex distributions showed that significantly more males (*n* = 23) were in the CU group as compared to the nCU group [*n* = 20; χ^2^(df = 1) = 4.69, *p* = 0.03].

We also compared schizotypy sub-scale scores to a previous normative sample (17) via calculations of Cohen’s *d* (64) with values of ±0.2/±0.5/±0.8 being indicative of a small/medium/large effect size, respectively. As can be seen from Table 1, schizotypy values were largely comparable to normative data, as no large effect sizes were found. A medium effect size was indicated for UnEx, with higher values in the normative sample as compared to the current sample (see Table 1).

**Table 1:** **Means, SDs, and effect sizes (Cohen’s *d*), comparing the values of the normative sample with the current sample**.

Questionnaire	Norm values (*N* = 508)		Current sample (*N* = 83)		Cohen’s *d*
	Mean	SD	Mean	SD	
O-LIFE: UnEx^a^	9.70	6.70	6.36	4.92	0.52
O-LIFE: CogDis^b^	11.60	5.80	10.61	5.28	0.17

*^a^Unusual experiences*.

*^b^Cognitive disorganization*.

As can be seen from Table 2, the groups (CU, nCU) were comparable in age. However, CU as compared to nCU scored higher on UnEx (as a trend), nicotine, cannabis, and alcohol use severity (since nCU were screened for weekly cannabis and cigarette use, the comparisons between nCU and CU were not conducted for these variables).

**Table 2:** **Age, schizotypy, and drug use statistics comparing CU and nCU**.

Variables	CU^c^ (*N* = 35)		nCU^d^ (*N* = 48)		*t*	*p*
	Mean	SD	Mean	SD		
Age	22.51	5.63	21.67	3.56	0.84	0.40
UnEx^a^	7.63	6.04	5.44	3.71	1.90	**0.06**
CogDis^b^	10.46	5.75	10.73	4.97	−0.23	0.82
Cigarettes/week	24.66	28.67	n/a	n/a	n/a	n/a
Joints/week	11.14	12.16	n/a	n/a	n/a	n/a
Nicotine use severity	1.94	1.86	0.08	0.45	5.78	**0.00**
Cannabis use severity	2.97	1.95	0.00	0.00	9.03	**0.00**
Alcohol use severity	2.29	1.84	1.31	1.42	2.72	**0.01**

*^a^Unusual experiences*.

*^b^Cognitive disorganization*.

*^c^Cannabis users*.

*^d^Cannabis non-users*.

*Values were compared between groups (CU, nCU) using independent *t*-tests (statistical results are shown in this table; *t*-values, df = 81, *p*-values). Significant group differences are highlighted in bold, trends in gray*.

### Cognitive functioning

#### Group comparisons

The separate univariate ANOVAs on the outcome measures revealed that CU performed significantly worse in the story-recall task, and slightly worse on the working memory task as compared to nCU (Table 3). The results for the remaining outcome variables were not significant (Table 3).

**Table 3:** **Descriptive and statistical values for the cognitive measures, comparing performance of CU and nCU**.

Variables	CU^b^ (*N* = 35)		nCU^c^ (*N* = 48)		*F*(1,81)	*p*	Partial η^2^
	Mean	SD	Mean	SD
Two-back % target correct	86.00	13.49	90.52	8.07	3.62	**0.06**	0.04
Two-back mean RT	822.57	164.00	821.42	205.92	0.00	0.98	<0.01
TMT^a^ index	23.77	13.58	23.56	16.58	0.00	0.95	<0.01
Story-recall % correct	55.90	14.78	67.21	12.19	14.55	**<0.01**	0.15

*^a^Trail making task*.

*^b^Cannabis users*.

*^c^Cannabis non-users*.

*Values were compared with univariate ANOVAs, and significant values are highlighted in bold, trends in gray*.

#### Regression analyses

The initial correlation analyses between task performances, schizotypy sub-scale scores, age, and drug measures revealed that neither age nor schizotypy related to cognitive functioning (all *p*-values >0.10, see Table 4). Accordingly, age was not further considered. UnEx and CogDis on the other hand were included as *a priori* predictions were formulated based on the published literature (see Introduction and Data Analysis).

**Table 4:** **Correlations between potential predictor variables and cognitive measures**.

Variables	Two-back task % target correct	Two-back task mean RT	TMT^c^ index	Story-recall % correct
Age	−0.06	−0.16	−0.08	−0.03
UnEx^a^	−0.08	0.06	0.08	−0.03
CogDis^b^	0.02	−0.01	−0.04	−0.07
Nicotine use severity	−0.24*	0.08	0.04	−0.41***
Cannabis use severity	−0.27*	0.14	0.10	−0.47***
Alcohol use severity	−0.29**	0.40***	0.28**	−0.07

*^a^Unusual experiences*.

*^b^Cognitive disorganization*.

*^c^Trail making task*.

*Significant at *p* ≤ 0.05.

**Significant at *p* ≤ 0.01.

***Significant at *p* ≤ 0.001.

*Significant values are highlighted in bold*.

The significant results from the subsequent regression analyses (see Data Analysis for further details) can be seen in Table 5. With regard to the control variables, we found that sex predicted verbal short-term memory. *Post hoc* independent *t*-tests revealed that women were significantly better than men in the story-recall task [women: 66.63 ± 12.08%, men: *m* = 58.54 ± 15.39%; *t*(81) = 2.65, *p* = 0.01]. Entering schizotypy in the second step explained no additional variance on top of sex (see Table 5). Drug use severity in the third step predicted significant amounts of variance in the outcome measures. Here, higher alcohol use severity predicted lower working memory performance, and higher cannabis use severity predicted reduced verbal short-term memory on top of sex and schizotypy (Table 5).

**Table 5:** **Significant results (including trends in gray) from the regression analyses assessing the effect of sex (step 1), schizotypy (UnEx^a^, CogDis^b^; step 2), and drug use severity (nicotine, alcohol, and cannabis; step 3) on cognitive measures**.

Outcome variables	Step	Significant predictor	β-value	Total *R*^2^	Δ*R*^2^	*F* for Δ*R*^2^
Two-back % target correct	3	Alcohol	−0.25*	0.15**	0.14**	4.25**
Two-back mean RT	1	Sex	−0.21^†^	0.04^†^	0.04^†^	3.61^†^
	3	Alcohol	0.39***	0.18**	0.13**	4.03**
Story-recall % correct	1	Sex	0.28**	0.08**	0.08**	7.02**
	3	Cannabis	−0.41**	0.30***	0.21***	7.46***

*^†^*p* ≤ 0.10*.

*Significant at *p* ≤ 0.05.

**Significant at *p* ≤ 0.01.

***Significant at *p* ≤ 0.001.

*^a^Unusual experiences*.

*^b^Cognitive disorganization*.

## Discussion

We investigated whether pure cannabis use hampers cognitive performance, or whether cognitive attenuation is also, or even better explained by associated licit drug use and psychotic-like features (schizotypy). For this purpose, we tested cognitive functions commonly associated with drug use and schizotypy in CU and nCU. The main findings were that (i) CU as compared to nCU performed worse on story recall and slightly worse on the two-back task, but not on the TMT, (ii) CU scored higher than nCU on positive schizotypy (as a trend), and drug use other than cannabis, (iii) regression analyses showed that enhanced cannabis use predicted decreased verbal short-term memory, whereas enhanced alcohol use predicted reduced working memory performance, (iv) none of the schizotypy sub-scales explained any additional variance in cognitive functioning. The implications of these findings are discussed below.

### Role of cannabis use severity

Our results showed that CU performed worse than nCU on tasks measuring verbal short-term memory (story recall), and higher cannabis use severity was associated with worse performance in this task. Our results also showed that these relatively negative cognitive implications were not associated with individuals’ self-reported schizotypy. The observation that worse story recall is associated with cannabis use is in line with previous studies (3, 13, 46). However, story recall (verbal memory) was the only task that was affected by cannabis use, whereas relatively impaired performance on another cognitive task (working memory as assessed with the two-back task) was related to enhanced alcohol use instead. Previous studies have indicated that cannabis use has a negative impact on working memory performance (46, 65) and mental flexibility (3, 66) as well. Our findings suggest that these previous findings on cannabis use were potentially confounded by concomitant non-excessive alcohol use (3, 67).

Despite some evidence that cannabis use is still associated with cognitive impairments after adjusting for alcohol use (68), independent studies (8, 36) report that CU tend to consume higher amounts of other drugs as well. This additional drug use, as frequently not assessed, might lead to misleading conclusions about cannabis effects on cognition. Particularly licit drug use like alcohol seems to be a relevant confounding factor. For instance, whereas in some studies alcohol use is either statistically controlled for (63) or subjects with alcohol abuse are excluded from participating (13, 14), other studies do not account for this variable (24, 69, 70, 71). Moreover, alcohol and cannabis are thought to exert comparable effects on cognition, i.e., cognitive attenuation in verbal memory (72–74), cognitive flexibility (75, 76), and working memory (77–79). Future studies should consider (non-excessive) licit drug use as a potential confounding factor when investigating the effects of cannabis use on cognition.

### Role of schizotypy

Of additional significance was the observation that schizotypy did not explain variance in most cognitive tasks. We do not think that this finding can be explained by deviant features of our sample, because we replicated many previous observations, i.e., that CU as compared to nCU scored slightly higher on measures of positive schizotypy (24, 27, 28, 41, 42, 44). The observation that schizotypy was not importantly related to cognitive functioning would indicate that the impairments in, e.g., working memory (19, 20), cognitive flexibility (21), and verbal memory (22, 23) may be influenced by individuals’ concomitant drug use.

Unfortunately, the above mentioned studies did not report on drug use (19, 21–23, 80), or only screened for substance use history without specifying the substances controlled for (20, 81). It is thus possible that substances (e.g., illicit as well as licit) influenced the relationship between schizotypal symptoms and the cognitive functions assessed in these experiments (38–40). In particular, our results suggest that cannabis may be more relevant than schizotypy for cognitive attenuations in verbal short-term memory, and alcohol may be more relevant than schizotypy for cognitive attenuations in working memory (82).

The specific cannabis effects on story recall, but not on the two-back or TMT may also suggest that not all cognitive functions are equally sensitive to cannabis-related attenuations. Even though many studies observe CU to show impairments compared to nCU on tasks measuring working memory (46) and mental flexibility (3, 66), this may not always be the case (71, 83). In fact, different meta-analyses draw inconsistent conclusions about which cognitive functions qualify as cognitive markers, or endophenotypes for pathological changes. Findings are inconsistent in CU and along the schizophrenia spectrum, with some studies pointing to verbal memory impairments in both populations (65, 84–86), some pointing to cognitive flexibility impairments (86, 87), and others reporting consistent working memory impairments in both patients with psychosis and CU (65, 85, 86). Alternatively, higher THC-content of used cannabis may relate to more prominent cognitive attenuations (88). Therefore, future studies should report the type and/or strength of cannabis used to improve reliability of findings.

Admittedly, all these complex functions tap into a variety of cognitive sub-functions. For this reason, to increase reliability of findings across studies and populations, the research community might consider behavioral markers that are less complex in their cognitive demands (89–92). Additionally, the pathophysiology of psychotic disorders is currently unknown, and the disorders are quite heterogeneous in their phenotypic expression. Consequently, we may increase the reliability of findings by accounting for seemingly related as well as unrelated factors potentially influencing the relationship between cannabis, cognition, and psychosis (-risk). For instance, studies could consider different yet potentially equally relevant personality traits such as those tapping on the autism spectrum (93) or the bipolar spectrum (94, 95). Beyond personality, studies could consider genetic predisposition (96), IQ (97), and neurochemical peculiarities such as dopamine receptor availability (98, 99) that may influence the effect cannabis exerts on cognition. Such factors are also relevant for the link between psychosis and drug use, e.g., genetic predisposition (100, 101), IQ (102–104), and neurochemical peculiarities (99). At present, it is impossible to account for all putatively influential variables, and hence additional studies need to be conducted to replicate our and similar findings, be it clinical, experimental, and/or epidemiological studies.

### Study limitations and future research

In the catchment area of our study, the “binge drinking culture” reflects on the high acceptance for alcohol use (105). Consequently, we refrained from pre-selecting participants according to their alcohol use, as the recruitment of pure CU (rather than polydrug users) turned out to be challenging, and was not facilitated by the modest incentives we could offer. Likewise, controlling for the co-use of nicotine seemed even more unavoidable, because cannabis is mostly used in combination with nicotine (106). Yet, controlling for nicotine could have been relevant, because nicotine itself might counteract the effects of cannabis on cognition (33, 107–109). We therefore suggest that future studies should elucidate the role of nicotine and cannabis more directly.

The gender composition differed between groups, a difference common to studies such as the current one. This gender difference could have also affected the group differences in story recall. Typically, females perform better on verbal short-term memory tasks than males (110), a finding also observed here. Since the nCU group consisted of more females than the CU, this group difference could alternatively explain the worse story recall in CU. However, since cannabis use related to worse story-recall performance on top of sex in the regression analysis, we deem it unlikely that the group differences are solely due to effects associated with the unequal sex distribution. Nevertheless, future studies on drug use and cognition should aim to control for sex differences.

A final, frequently mentioned study limitation is the sample size, also relevant to the conducted analyses. For regression analyses, the guidelines for recommended sample sizes vary, from using 50 + 8× *N* variables (111–113) to 10 participants per predictor variable (114). Obviously, a larger sample size would always be advisable. Yet, our sample size matches sample sizes in other studies reporting on preselected minority samples of (relatively) pure drug users (3). A potential reason could be firstly, that these individuals are either extremely difficult to motivate, or secondly, that pure users of drugs are a rarity, at least in our study region. The difficulty of finding pure CU may also be reflected in population descriptions over the last 30 years; many studies inferred on the influence of cannabis use on cognition and mental health risk without necessarily ensuring that individuals did not also consume other licit and illicit drugs. We thus face the future challenge to disentangle the impact of a specific drug use or synergetic drug uses on cognition and mental health (115–117).

Finally, cognitive attenuations related to cannabis use seem more overt in heavy as compared to moderate or light users (14, 118, 119). A higher frequency of cannabis use in our sample might have exacerbated the reported cognitive attenuations. Though definitions for heavy use may vary (118, 119), the frequency of cannabis use (joints/week) in our sample seems to indicate heavy use according to a previous report on pure CU (63). To note, our data point to a negligible influence of frequency of pure cannabis use (see Materials and Methods).

## Conclusion

While pure cannabis and alcohol use seem associated with adverse effects on cognition, other risk factors (e.g., nicotine use) might also be relevant. Schizotypy, on the other hand, seems unrelated to cognitive attenuation. Results stress the importance to control for additional substance use (and non-excessive use in particular), whether illicit or licit, when assessing the effect of schizotypal symptoms and/or cannabis use on cognition. Moreover, heterogeneity of cannabis-related attenuations of specific cognitive functions may be avoided by controlling for additional factors potentially influencing the relationship between cannabis, cognition, and psychosis (-risk).

## Author Contributions

All authors significantly contributed to the conception or design of the work, the analysis, and interpretation of data for the work, as well as revising it critically for important intellectual content. Dr. Daniela A. Herzig was additionally involved in the data acquisition and drafting the first version of the manuscript. All authors approve of the final version of the manuscript, therefore, ensuring that questions related to the accuracy or integrity of any part of the work are appropriately investigated and resolved.

## Conflict of Interest Statement

The authors declare that the research was conducted in the absence of any commercial or financial relationships that could be construed as a potential conflict of interest.
